# Reduced striatal dopamine release during motor skill acquisition in Parkinson’s disease

**DOI:** 10.1371/journal.pone.0196661

**Published:** 2018-05-30

**Authors:** Shoji Kawashima, Yoshino Ueki, Takashi Kato, Kengo Ito, Noriyuki Matsukawa

**Affiliations:** 1 Department of Neurology and Neuroscience, Nagoya City University Graduate School of Medical Science, Mizuho-ku, Nagoya, Japan; 2 Department of Rehabilitation Medicine, Nagoya City University Graduate School of Medical Science, Mizuho-ku, Nagoya, Japan; 3 Department of Brain Science and Molecular Imaging, Research Institute, National Center for Geriatrics and Gerontology, Morioka, Obu, Aichi Prefecture, Japan; University of Florida, UNITED STATES

## Abstract

**Background:**

Striatal dopamine is functionally important for the acquisition of motor skills. However, it remains controversial as to whether intrinsic processing of motor learning is impaired in patients with Parkinson’s disease (PD), and if yes, whether the impairment is associated with altered striatal dopamine release. Additionally, most neuro-imaging studies of patients with PD have focused on motor sequence learning. In contrast, skill acquisition, specifically, the reconstruction of muscle control of isolated movements, has barely been studied.

**Method:**

In this study, we used a repetitive skill training task to measure the peak acceleration of left thumb movement during a process to achieve fine tuning of motor skill. Using ^11^C-raclopride (RAC) positron emission tomography, we investigated changes in striatal dopamine levels in two conditions of a skill acquisition task: initial skill training (Day 1) and acquired condition (Day 2) with eight patients with PD and age-matched healthy subjects (HS).

**Result:**

In HS, the mean acceleration of each session improved through repeated training sessions on Day 1. However, in patients with PD, the training-associated increase was less than that for HS, and this suggests that repetitive skill training does not result in the effective improvement of motor performance. The regions of interest (ROI) analysis revealed that the RAC-binding potential (BP) was significantly reduced in the right putamen on Day 1 compared with Day 2 in HS. In patients with PD, BP within the right putamen was unchanged. Further, we found that patients with PD had increased dopamine levels within the right ventral striatum (VST) and right caudate (CAU) on Day 2, which was greater than that in HS. These results suggest the impaired activation of the putamen during skill acquisition in patients with PD and compensated hyperactivation of the VST and CAU for the reduced dopamine release within the dorsal putamen (DPU).

**Conclusion:**

Our findings suggest that patients with PD had insufficiency in the process to improve motor skills. Different patterns of striatal dopamine release are relevant to the impairment of these motor functions in patients with PD, at the early stage of the disease.

## Introduction

Striatal dopamine depletion due to degeneration of the nigrostriatal dopaminergic neuron causes motor disturbances in patients with Parkinson’s disease (PD). In the nigrostriatal pathway, the motor cortical areas project major glutamatergic fibers into the striatum, which belongs to a series of basal ganglia-thalamo-cortical loops that project back to the motor cortex via the motor thalamic nucleus [1, 2]. Disruption of these loops causes the various motor signs associated with PD [3–5]. Some studies have observed the impairment of motor learning in patients with PD [5–9]. An animal study reported that impairment of motor learning was recovered after the administration of L-DOPA [10].

Acquisition of motor skill is manifested by increased accuracy or speed of performance due to repeated exposure to a specific procedure, without conscious recollection of the prior learning episode or the rules underlying the task [11]. Acquisition of motor skill has distinct stages, which are associated with dynamic changes in motor representation [12–15]. At the initial skill acquisition stage, consisting of several training sessions that range in duration from minutes to hours, rapid improvements in motor performance are generally observed [16–19]. At the acquired stage, the motor skill is carried out effortlessly, with minimal attentional resources [13, 20]. Many functional imaging studies have revealed that the neural basis of motor learning is attributable to different portions of the brain, including the motor cortices, cerebellum, and basal ganglia, depending on the learning stage [21–25].

Dopaminergic signals in the striatum and motor cortex play essential roles in sequential motor learning [26]. To elucidate these processes in humans, ^11^C-raclopride (RAC) positron emission tomography (PET) was used in this study [27]. Several studies have reported impairment of sequential motor learning in patients with PD. On the contrary, others have reported preserved motor skill acquisition in these patients [28–30]. Additionally, little is known about the relationship between striatal dopamine and the intrinsic process of motor skill acquisition in patients with PD.

Recently, we reported that striatal dopamine release is related to the intrinsic processing of new motor memory during skill acquisition in humans [31]. The aim of the present study was to clarify whether patients with PD have different patterns of change in striatal dopamine during the process of motor skill acquisition. To evaluate this, we investigated changes in dopamine levels in the striatum, as related to the intrinsic processing of skill acquisition between different stages, using a marker of D2/D3-receptor binding with RAC-PET in patients with PD and aged healthy subjects (HS) [32]. Our hypothesis was that the impairment of striatal dopamine release in patients with PD was associated with the dysfunction of motor skill acquisition and retention of the trained skill.

## Methods

### Subjects

A total of eight HS (mean age ± SD: 68.7 ± 2.8 years) and eight patients with PD (mean age ± SD: 66.9 ± 3.5 years) were enrolled in the study. All subjects were right-handed according to the Edinburgh Inventory [33], without a history of any other neurological or psychiatric disorders, or orthopedic issues. All patients with PD fulfilled the UK Brain Bank Criteria for the clinical diagnosis of PD, and corresponded to categories two or three of the Hoehn and Yahr Scale [34] in their ‘off-medication’ state. The patients were studied after 12 h overnight off-medication with levodopa replacement. Motor function in the patients, both off and on medication, was assessed according to the motor section of the Unified Parkinson’s Disease Rating Scale (UPDRS) [35]. All subjects provided written informed consent to the study, in accordance with the dictates of the ethics committee of the Clinical Research Management Center of Nagoya-City University Hospitals and the research ethics committee of the National Center for Geriatrics and Gerontology.

### Neuropsychological test

To compare baseline profiles, we tested the MMSE for the assessment of global cognition. In this study, patients with less than 24 points of MMSE were excluded. The visuoperceptual ability and attention were tested with the Trail Making Test Part A (TMT-A), and rapid-set shifting and visuoperceptual ability were tested with the Trail Making Test Part B (TMT-B). The result of TMT partially depended on hand motor speed. To minimize the differences in hand motor speed and visuoperceptual ability between the groups, we calculated the TMT B-A by subtraction of the time to complete TMT-B from TMT-A [36]. The TMT B-A provided a relatively pure indicator of executive function.

### Experimental procedure

The task used in the study was modified from the motor skill acquisition protocol developed by Muellbacher et al. [37, 38]. Subjects practiced rapid contraction of their left thumb to the beat of a metronome every 3 s, and learned how to accelerate their left thumb appropriately. They were then asked to perform the metronome-paced movement as quickly as possible, with the aid of visual feedback. Change in the sequential one-dimensional acceleration of the subjects’ left thumb during each contraction was recorded with a piezoelectric accelerometer mounted on the proximal phalanx.

**Fig 1:**
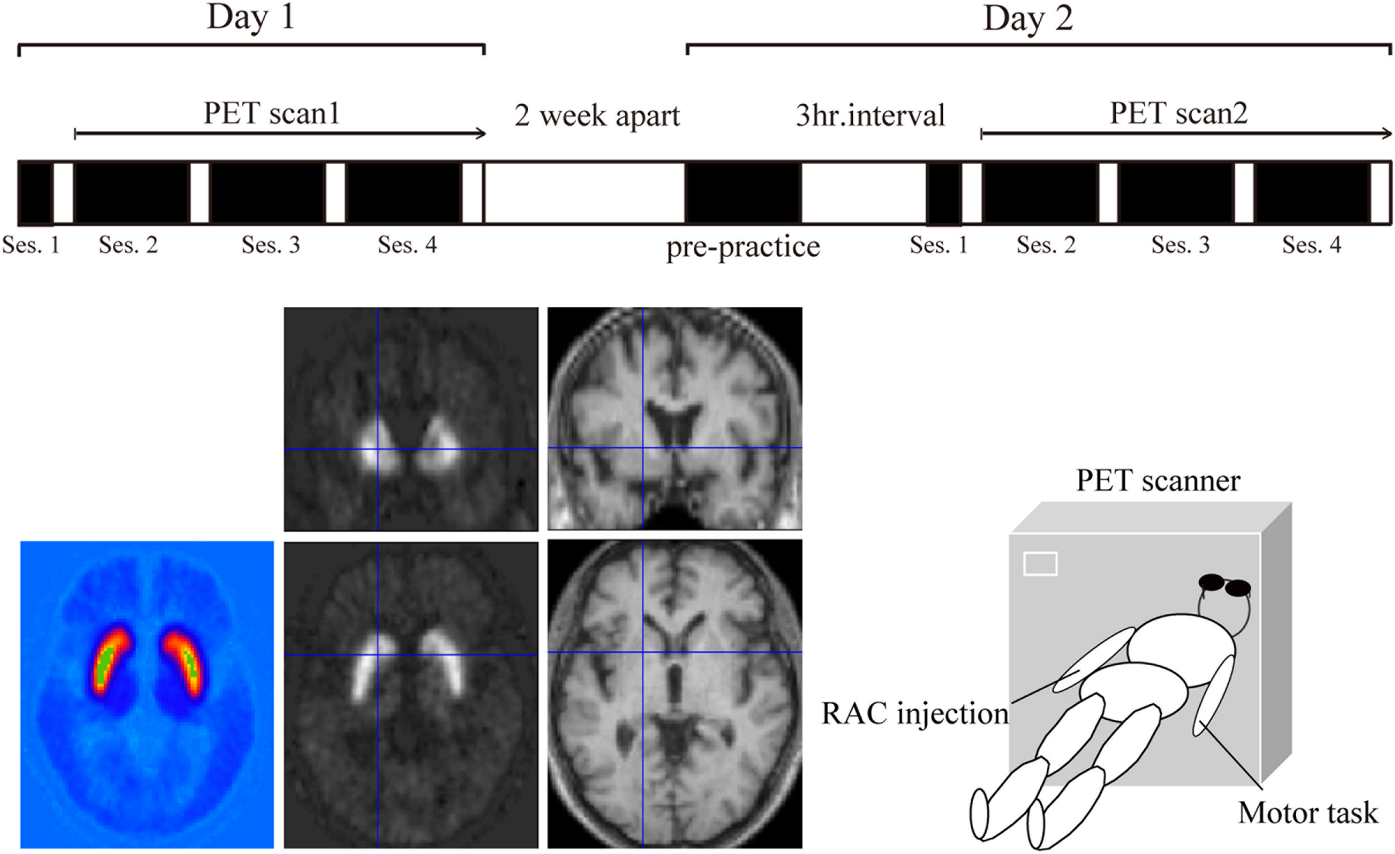
Experimental protocol. The upper panel shows the overview of experimental protocol. In the initial skill-training condition (Day 1), subjects performed one session of motor practice inside the scanner (session 1: 60 movements), and then completed three further sessions (sessions 2–4: 240 movements) while undergoing PET scanning. In the acquired condition (Day 2), 2 weeks later, subjects performed four blocks of a pre-practice task (block 1: 60 movements, blocks 2–4: 120 movements) 3 h before the PET scan, and then performed the motor task again in a manner identical to the initial skill-training condition (session 1: 60 movements, sessions 2–4: 240 movements). The lower-right panel illustrates data collection during PET scanning. The injection of RAC was performed into the subjects’ peripheral vein on their right forearm by an expert radiologist of the nuclear medicine. The computational motor task was performed on the subjects’ left hand by a neurologist. Subjects practiced rapid contraction of their left thumb to the beat of a metronome every 3 s, and learned how to accelerate their left thumb appropriately. They were then asked to perform the metronome-paced movement as quickly as possible, with the aid of visual feedback. The lower-left panel shows a sample image of RAC-BP and MRI T1. The RAC-BP indicated radioactivity concentration of the dopamine receptor. (A) RAC-BP image which was calculated using PMOD software (PMOD Technologies, Zurich, Switzerland). (B) Coronal and axial view of the co-registered RAC-BP image by using SPM5 software (Department of Imaging Neuroscience; freely available at http://www.fil.ion.ucl.ac.uk/spm). (C) The MRI T1 image for the registration with PET images.

Subjects underwent a structural MRI for registration with PET images. To evaluate potential changes in striatal dopamine release associated with different stages of motor skill training, RAC-PET scans were obtained twice, on separate days at least 14 days apart (Day 1: initial skill training condition, Day 2: acquired condition). On Day 1, subjects performed one session of motor practice (session 1: 60 movements), and then completed a further three sessions (sessions 2–4: 240 movements) while undergoing PET scanning. On Day 2, subjects performed a pre-practice session 3 h before the PET scan, and then performed the motor practice again in a manner identical to that performed on Day 1 (Fig 1).

### Behavioral data analysis

For the analysis of motor performance, mean peak acceleration of the 60 (session 1) or 240 (sessions 2–4) movements was calculated and expressed in cm/s^2^. Mean acceleration during session 1 on Day 1 was defined as the baseline. To compare the difference in baseline motor performance between groups, the mean acceleration of baseline was compared using the *t*-test. Additionally, the ratio of behavioral change against baseline, the percentage change was calculated as [(mean peak acceleration in session X − baseline) / baseline] × 100. A repeated measurement in analysis of variance (ANOVA) (conditions: Day1 and Day2, group: PD and HS) was used to compare group difference in the changes of the mean acceleration of sessions 1–4. Additionally, due to the group difference in baseline motor performance, we performed the within-group comparisons in separate groups using one-way ANOVA for each condition. In the analysis, we compared the mean acceleration for sessions 1–3, because patients with PD had poor motor performance that caused a motor performance decline in the last session 4. To compare the effect of motor skill training, the mean accelerations in session 1 on Day2 and Day1 were compared using the *t*-test.

In addition, Pearson’s correlation analysis was performed to investigate the association between the mean acceleration during session 1 (baseline) and the score of UPDRS part 3 in patients with PD.

All statistical analyses were performed using SPSS version 22.0 for Windows (IBM Japan, Tokyo, Japan), and *P* values ≤ 0.05 were considered significant.

### Imaging data acquisition and analysis

The detail of the imaging data acquisition and the protocol used for PET are reported in our previous report [31]. Using regional time-activity curves, the RAC binding potential (BP), which indicated changes in radioactivity concentration, was computed. The RAC-BP reduction between the two conditions reflects endogenous dopaminergic transmission related to the difference in task performance during the PET scan, from just before the injection of RAC until several hours afterwards [39]. Lower BP indicates greater dopamine release (increased dopamine occupies more dopamine receptors, and leaves fewer available receptors for RAC to bind to).

On each subject’s MRI T1 image for all planes, ROI were bilaterally traced around the putamen and caudate. Sphere reference regions were traced on the bilateral cerebellum as regions with a 10 mm radius placed over the cerebellar hemisphere. These ROI and reference regions were defined three-dimensionally in each hemisphere, and located at reproducible anatomical positions for all subjects. RAC-BP, which indicated changes in radioactivity concentration, was calculated from the radioactivity concentration ratios in receptor-rich regions (bilateral putamen and caudate) and receptor-less (cerebellum) regions for both conditions. Each BP was calculated using a Logan reference-region graphical analysis [40].

The changes in RAC-BP on Days 1 versus 2 were analyzed separately for both groups using the two-tailed paired samples *t*-test. To compare the difference in the ratio of change in RAC-BP, we calculated the percentage change in RAC-BP in the putamen and caudate, as described previously [41]. The percentage change in RAC-BP was defined as [(BP-Day 2 − BP-Day 1) / BP-Day 2] × 100. The group differences in each ROI were examined using the paired *t*-test.

Moreover, to investigate the difference of RAC-BP concerning functional subdivision of the putamen, we performed ROI analysis separately for the dorsal putamen (DPU), ventral striatum (VST), and caudate (CAU) based on a previously described method [42, 43]. RAC-BP was calculated from the radioactivity concentration ratios in receptor-rich regions (bilateral VST, DPU, CAU) and receptor-less (cerebellum) regions, using a Logan reference-region graphical analysis. In all statistical analyses, the threshold of significance was set at *P* < 0.05.

## Results

### Behavior

The baseline profiles of patients with PD are summarized in Table 1. The patients were in the early stage of PD, with mild clinical disability (mean disease duration 4.0 ± 2.5 years; Hoehn and Yahr stage 1.6 ± 0.5). Group comparisons of the profiles are summarized in Table 2. There were no statistical differences in global cognition and executive function, which was tested by the TMT B-A. Additionally, there was no correlation between the mean acceleration of baseline and UPDRS motor score.

**Table 1:** Clinical characteristics of the patients with Parkinson’s disease.

	Age (years)	Duration (years)	HY stage	UPDRS motor	Laterality	LEDD (mg)
Case- 1 (female)	67	2	1	8	Left	201
Case 2 (female)	68	5	2	7	Left	125
Case 3 (male)	62	3	2	9	Right	100
Case 4 (female)	61	1	1	4	Right	380
Case 5 (male)	61	4	1	9	Left	220
Case 6 (male)	65	7	2	12	Right	150
Case 7 (male)	78	2	2	12	Left	300
Case 8 (male)	65	8	2	22	Right	702
Mean ± SD	65.9 ± 5.6	4 ± 2.5	1.6 ± 0.5	10.3 ± 5.4		272 ± 197

Duration: Disease duration from onset, HY stage: Hoehn and Yahr stage, UPDRS motor: motor sections of united PD rating scale, LEDD: l-dopa equivalent daily dose. Calculation of LEDD for each patient was based on the theoretical equivalence to l-dopa as follows: l-dopa dose + l-dopa dose × 1/3 [if on entacapone + bromocriptine (mg) × 10 + cabergoline or pramipexole (mg) × 67 + ropinirole (mg) × 20 + pergolide (mg) × 100 + apomorphine (mg) × 8].

**Table 2:** Baseline characteristics between healthy subjects and patients with Parkinson’s disease.

	Healthy subjects	Parkinson’s disease patients	*P* value
Gender	5 males, 3 females	5 males, 3 females	N.S.
Age	68.7 ± 2.8	65.9 ± 5.6	N.S.
MMSE	28.2 ± 1.1	28.8 ± 0.6	N.S.
TMT B-A	57.5 ± 22.6	73.9 ± 58.1	N.S.

MMSE: mini mental scale examination, TMT B-A: Trail Making Test Part B-Part A (second)

N.S.: not significant

**Fig 2:**
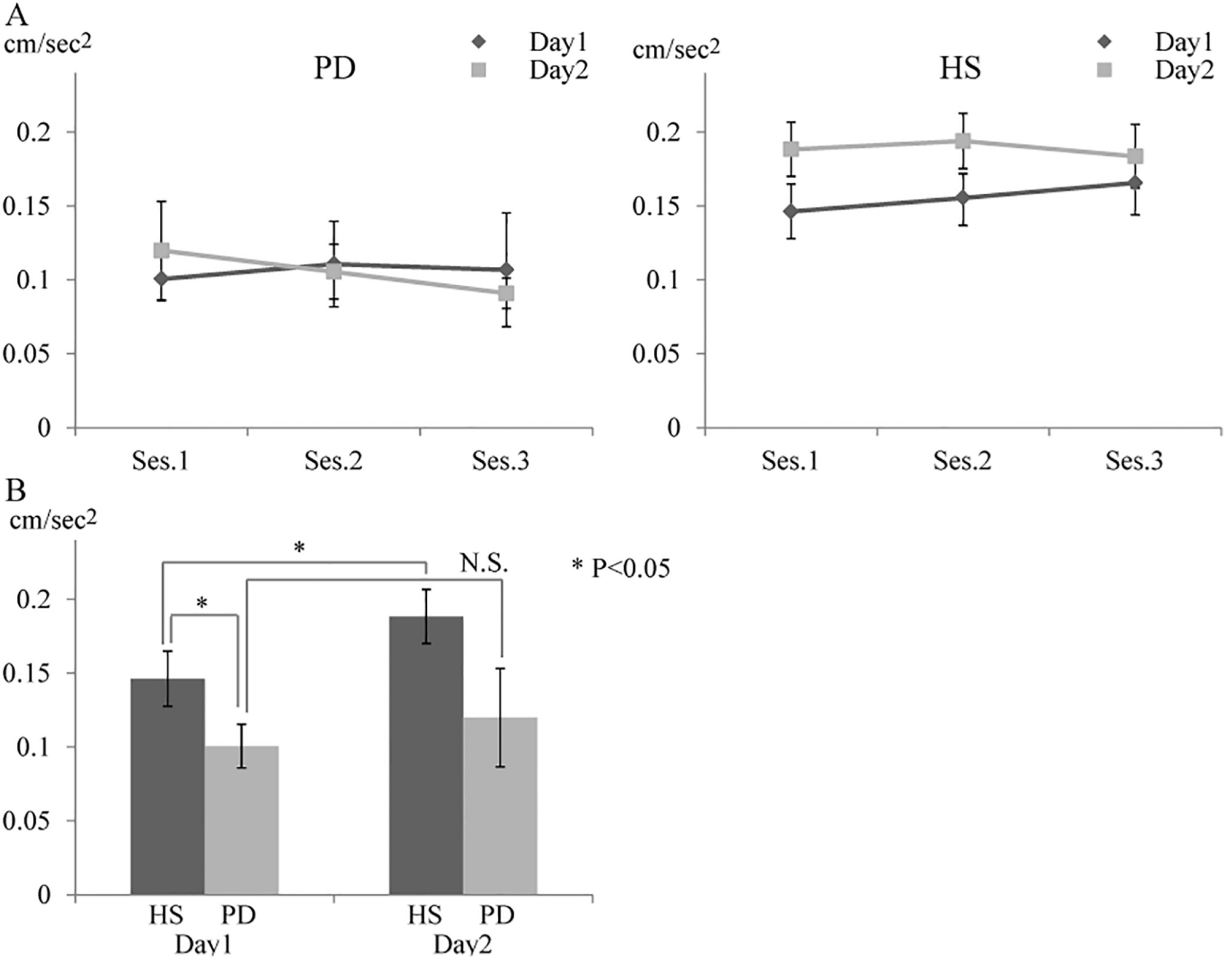
Mean acceleration changes of healthy subjects (HS) and patients with Parkinson’s disease (PD). (A) The graph shows the changes of mean acceleration of sessions 1–3 in patients with PD (left figure) and HS (right figure). (B) The graph shows the changes of the mean acceleration in session 1 between Day1 and Day2. Compared with the initial skill-training (Day 1), HS group showed significant increase of the mean acceleration in Day2. In PD group, the mean acceleration was not significantly changed in Day2.

Between groups, there was a significant difference in the mean acceleration of baseline (PD: 0.101 ± 0.015 cm/s^2^ vs. HS: 0.149 ± 0.052 cm/s^2^; *P* < 0.05). (Fig 2B) In each group, the changes in the mean acceleration of sessions 1–4 on Day1 (initial skill training condition) were calculated using the change ratio against baseline motor performance. HS group showed a gradual increase in mean accelerations until session 3 (percentage change was 9.9% in session 2, 21.2% in session 3, and 20.9% in session 4). However, the PD group showed just a slight increase in session 2 and then showed a remarkable decrease (percentage change was 10.0% in session 2, 6.1% in session 3, and −14.1% in session 4).

A repeated measurement in ANOVA (conditions with two levels, group with two levels) was performed to investigate the group difference in the changes of the mean acceleration of sessions 1–4. A significant main effect was demonstrated for group (*P* < 0.001, F[1, 30] = 38.7), and for condition (*P* = 0.025, F[1, 30] = 8.1). But there was no effect for group * condition (*P* = 0.076, F[1, 30] = 4.6). Considering the remarkable group difference between the baseline motor performance and the performance decline in the patients with PD, the behavioral analysis was limited to sessions 1–3, and the within-group comparisons to search the differences in motor skill acquisition were performed separately for each group, using the one-way ANOVA. In within-group comparisons on Day 1, HS group demonstrated a significant main effect of time (*P* = 0.04, F[3] = 4.1), however in patients with PD, no effect of time was demonstrated (*P* = 0.14, F[3] = 2.2). In within-group comparisons on Day 2, there were no significant effects of time in both groups (*P* = 0.96 in the HS group; *P* = 0.34 in the PD group). In the comparisons of the mean acceleration in session 1 on Day2 against Day1, HS group showed significant increase of the mean acceleration in Day2 (Day 1: 0.149 ± 0.019 cm/s^2^ vs. Day 2: 0.188 ± 0.018 cm/s^2^; P < 0.05). In PD group, the mean acceleration was not significantly changed. (Fig 2)

These different patterns of the change in accelerations suggested that motor performance was improved through repeated motor training in HS; however, such an effect of training was disturbed in patients with PD.

### ^11^C-raclopride PET

In comparisons of the percentage change in RAC-BP within the right putamen, HS showed significantly greater increases in percent change than that in patients with PD (16.7% ± 14.9% vs. 0.03% ± 12.7%; *P* < 0.05). In contrast, there was no statistical difference within the right caudate (−0.01% ± 16.4% vs. 2.7% ± 17.2%; *P* = 0.75) and no differences within either the left putamen or left caudate.

In the three ROI analyses for two subdivisions within the putamen, HS showed that RAC-BP in the right DPU was reduced on Day 1 compared with Day 2 (2.11 ± 0.32 vs. 2.34 ± 0.61; *P* < 0.05). However, RAC-BP in the right VST was unchanged (1.92 ± 0.47 vs. 1.91 ± 0.31; *P* = 0.85). In patients with PD, RAC-BP in each ROI did not change significantly (DPU Day 1: 2.56 ± 0.61, Day 2: 2.67 ± 0.58, *P* = 0.50; VST Day 1: 1.67 ± 0.73, Day2: 1.54 ± 0.37, *P* = 0.43).

With regard to Day 1 (initial skill training), although it did not reach statistical significance, HS showed lower RAC-BP within the right DPU than patients with PD (2.11 ± 0.32 vs. 2.56 ± 0.61; *P* = 0.08). There was no statistically significant group difference in RAC-BP within the VST or CAU. In contrast, on Day 2 (acquired condition), RAC-BP of patients with PD within the right VST and CAU was lower than that of HS (VST HS, 1.91 ± 0.31, PD, 1.54 ± 0.37, *P* < 0.05; CAU HS, 2.29 ± 0.55, PD, 1.69 ± 0.55, *P* < 0.05; Table 3).

**Table 3:** ^11^C-raclopride binding potential (RAC-BP) of the three ROI within the right striatum in healthy subjects (HS) and patients with Parkinson’s disease (PD).

		Healthy subjects	Parkinson’s disease	*P* value
**Dorsal putamen**	BP Day 1	2.11 ± 0.32	2.56 ± 0.61	0.08
	BP Day 2	2.34 ± 0.29	2.67 ± 0.58	N.S.
**Ventral putamen**	BP Day 1	1.92 ± 0.47	1.67 ± 0.73	N.S.
	BP Day 2	1.91 ± 0.31	1.54 ± 0.37	<0.05*
**Caudate**	BP Day 1	2.17 ± 0.66	1.63 ± 0.56	0.09
	BP Day 2	2.29 ± 0.55	1.69 ± 0.55	<0.05*

BP Day 1: ^11^C-raclopride binding potential on Day 1, BP Day 2: ^11^C-raclopride binding potential on Day 2, N.S.: not significant,

* statistically significant

## Discussion

The goal of the study was to investigate changes in striatal dopamine levels measured with RAC-PET in HS and individuals with PD on different days of motor skill training: an initial skill training condition and an acquired condition. In general, motor learning is based on two aspects. The first is the process of forming complex movements with sequential elements (motor sequence learning), and the second is reconstructing muscle control of isolated movements (skill acquisition; encoding elementary aspects of movement) [44]. Most neuro-imaging studies of patients with PD, including RAC-PET, have focused on motor sequence learning. On the contrary, our experimental protocol was focused on the latter skill, i.e., skill acquisition.

The behavioral results of HS revealed a training-associated increase in the mean acceleration during initial skill training conditions (Day 1). Compared with the mean acceleration of sessions 1–3 on Day 1, HS showed a significant increase through repeated training sessions, until performance reached a maximum in session 3. However, in patients with PD, there was no significant change in the mean acceleration on Day 1. This result suggests that repetitive skill training does not result in the effective improvement of motor performance in patients with PD.

The ROI analyses clarified that patients with PD showed a significantly lower percent change within the right putamen (0.03%) than that shown by HS (16.7%). This indicates that putaminal dopamine release during motor skill acquisition is impaired in patients with PD. In addition, the results of the three ROI analyses suggest compensated hyperactivation of the ventral putamen and right caudate for reduced dopamine release within the dorsal putamen.

### Impaired skill acquisition in patients with PD

In the present study, patients with PD neither showed significant increase in mean acceleration across the sessions, nor in total acceleration between Day 1 and Day 2. There was no accompanying percentage change in RAC-BP within the putamen in patients with PD. These results suggest that fine-tuning of muscle control of isolated movements was impaired in PD, and that the impairment may be associated with dysfunction of striatal dopamine release during skill acquisition. Further, within the right DPU, which belongs to the sensorimotor striatum, HS showed lower RAC-BP compared with patients with PD. This is likely to be explained by impaired activation of the DPU during skill acquisition in patients with PD.

Recently, some evidences have been found to relate the relationship between the encoding elementary aspects of movement and striatal dopamine release. In animal studies, striatal regional administration of a D1 receptor antagonist was found to impair the acquisition and consolidation of motor skills, and a D2 receptor was also found to have an important role in motor learning [45, 46]. Dopaminergic signaling in the primary motor cortex is necessary for motor skill acquisition, but not for the execution of a learned task [26, 47]. In a previous study of patients with PD, some RAC-PET studies found that striatal dopamine plays a key role in different tasks or methodologies such as sequential finger movement, walking, and repetitive transcranial magnetic stimulation during rest and task production [40, 48]. With regard to the cortico-striatal connection, diffusion tensor imaging of fiber tracts has shown that the sensorimotor striatum connects mainly to the motor cortex, premotor cortex, supplementary motor area, and prefrontal cortex [49, 50]. Functional MRI studies have revealed that the cortico-striatal circuit contributes to the early stage of motor learning, that is, acquisition and consolidation of motor skill memory [21–25]. Moreover, previous studies have shown that dopamine selectively enhances active synapses in a task-specific manner to increase the signal-to-noise ratio [51, 52]. With regard to patients with PD, it has been reported that resting state functional connectivity involving the cortico-striatal circuit is altered [53, 54]. Therefore, reduced putaminal dopamine release would cause impairment in the motor cortico-striatal circuit activation during the task, leading to impaired skill acquisition.

Post-mortem evidence and neuroimaging studies have demonstrated that during the early stage of PD, the degenerative process targets dopaminergic fibers innervating the DPU contralateral to the clinically affected limbs, with milder reductions in dopamine in the ipsilateral putamen and the head of the caudate [55–57]. As the disease progresses, dopamine loss becomes significant in the ipsilateral striatum, rostral caudate, and finally, in the ventral putamen [56, 58]. In the present study, patients in the early stage of PD were recruited. It is likely that for these patients, the dopaminergic fibers innervating the DPU were starting to be pathologically affected. The dopamine decrease in the DPU during skill training may mainly reflect reduced dopamine release in this area caused by the pathological degeneration of dopaminergic presynaptic neurons.

### Dysfunction of the retention of acquired skill

Pendt et al. demonstrated that fine-tuning and retention of acquired skills was impaired in patients with PD [30], and as such the lack of training-associated increase in mean acceleration on Day 2 may suggest insufficient retention of acquired skills in patients with PD. Even though we recruited patients with mild clinical signs, motor signs affecting the upper extremities were likely to affect the behavioral outcomes of patients with PD. This phenomenon is known as the sequence effect; a progressive slowing in speed or progressive decrease in the amplitude of repetitive movements observed in patients with PD [59, 60]. The effect may not be caused by exhaustion only but may also be associated with freezing. This performance decline has also been observed in drug-naïve patients with PD during finger tapping and the repetitive movements involved in a pegboard task [61]. Considering these findings, the lesser increase in mean acceleration in patients with PD may be explained by both dysfunction of the retention of acquired skill and the sequence effect.

### Clinical implications

Although a number of studies, including this study, have investigated acquisition and retention of acquired skill in patients with PD, outcomes have varied widely. Nieuboer et al. reviewed studies that evaluated acquisition and retention in a broad range of tasks, and suggested that acquisition does occur in patients with PD, but performance on a task during acquisition is typically impaired relative to controls [62]. Another meta-analysis reached that same conclusion [63].

The controversy among studies may be explained by heterogeneity in the methodology and characteristics of the patient samples selected by the studies. First, with regard to motor performance and skill acquisition in patients with PD, it has thus far been difficult to separate internal processing of skill acquisition from expression of behavior, because motor learning can only be discerned by changes in performance and dopamine directly impacts performance. Thus, interpretation of the results of behaviorally impaired patients is influenced by task performance [64, 65]. In addition, as for the patient sample in this study, there were no group differences in global cognition or executive function. It suggested that the dysfunction of the acquisition and retention of the task was not primarily associated with the attention network between the frontal lobes and caudate but rather with the motor cortico-striatal circuit. We reported that patients with PD require greater activation to compensate for basal ganglia dysfunction while performing movements. In fact, in this study, ROI analysis of patients with PD showed an increase of dopamine within the right VST and right CAU that was greater than that in HS. Considering these findings, retention of a well-practiced movement may place patients with PD in a situation of keeping more attention and cause increased functional activation of these lesions, to compensate for dysfunction in the dopaminergic fibers innervating the DPU.

Additionally, as for the therapeutic effect of motor learning, human studies of healthy normal or stroke patients showed that dopamine administration can enhance the ability to encode an elementary motor memory in the primary motor cortex [66, 67]. In patients with PD, some research has reported that L-DOPA [68] or subthalamic nucleus deep brain stimulation [69] may improve motor skill learning. Evidence suggests that dopamine therapy directly improves impaired motor learning in patients with PD. Therefore, further study is needed to identify an effective motor training paradigm for rehabilitation.

### Limitations of the study

The present study had some limitations relating to its protocol design, which was based on group comparisons between two conditions during PET scanning. First, because of the small sample size, we could not find statistical differences using the voxel-wise analysis. Second, we did not investigate RAC-BP at rest in patients with PD because the purpose of this study was to evaluate striatal dopamine change during initial skill training compared to the acquired condition. Therefore, we analyzed changes in RAC-BP on Days 1 versus 2 separately in patients with PD and HS using the two-tailed paired samples *t*-test. However, patients with PD have an altered regulation of dopamine receptors in association with disease duration. A RAC-PET study demonstrated that patients with de novo drug-naïve PD showed an increase in RAC uptake in the putamen. Further, it clarified that the patients showed a reduction in RAC binding and returned to normal levels 3–5 years after the first PET [70]. In this study, the mean disease duration was 4.0 ± 2.5 years. Therefore, the baseline RAC-BP of patients with PD may have been at a normal level, and we estimated little group difference in baseline RAC-BP.

Third, the subjects in this study performed the task for a relatively long period of time (780 repetitive movements) compared with other studies that used functional MRI. Particularly on Day 2, they had to perform pre-practice before practice during PET. Such a long task might cause exhaustion in patients with PD, and it influenced the data variability. Data variability had reduced statistical power. Particularly in the last session 4, the mean acceleration of patients with PD was decreased to −14.1% from baseline. Considering the performance decline, the behavioral analysis was limited to sessions 1–3.

Fourth, there was a tendency that right putaminal dopamine release in patients with left-side dominance was lower than that in patients with right-side dominance on Day 1. Although it did not reach significance due to a small sample size, four patients with left-side dominance showed a slightly higher RAC-BP in the DPU than that of four patients with right-side dominance (2.87 ± 0.66 vs. 2.41 ± 0.71). It has been reported that the motor-symptom laterality affects acquisition in PD; that is, left-onset patients made more errors in feedback-based associative learning [71]. In this study, we thought that the difference in the dominant side was not directly affected by ROI analysis because we did not use the data comparing laterality of the dopamine release. However, it was possible that the motor-symptom laterality caused data variability in part. Therefore, the protocol of future studies on motor learning should consider motor-symptom laterality.

Although these limitations restricted our interpretation of the results, this study was original in that it aimed to investigate skill acquisition by measuring peak acceleration during a process to achieve fine tuning of a newly practiced motor skill. The findings of this study present a disease-associated difference in striatal dopamine change during motor skill training.

### Conclusions

We investigated changes in motor performance and striatal dopamine release on different days of skill training, using a task to fine tune muscle control. The results revealed that patients with PD have insufficiency in acquiring and repeating a motor skill. Different patterns of striatal dopamine release are relevant to the impairment of these functions, at the early stage of the disease.

## Abbreviations

ANOVA: analysis of variance
BP: binding potential
CAU: caudate
DPU: dorsal putamen
HS: healthy subjects
PD: Parkinson’s disease
PET: positron emission tomography
ROI: region of interest
VST: ventral striatum
